# The expression of p-ATF2 involved in the chondeocytes apoptosis of an endemic osteoarthritis, Kashin-Beck disease

**DOI:** 10.1186/1471-2474-14-209

**Published:** 2013-07-16

**Authors:** Jing Han, Xiong Guo, Wuhong Tan, Feng Zhang, Jiangtao Liu, Weizhuo Wang, Peng Xu, Mikko J Lammi

**Affiliations:** 1 Faculty of Public Health, College Medicine, Key Laboratory of Environment and Gene Related Diseases of Ministry Education, Key Laboratory of Trace elements and Endemic Diseases, Ministry of Health, Xi’an Jiaotong University, 710061 Xi’an, Shaanxi, PR China; 2 Department of Orthopedics Surgery, The Second Affiliated Hospital, College of Medicine,Xi’an Jiaotong University, 710061 Xi’an, Shaanxi, PR China; 3 Department of Orthopaedics Surgery, The Xi’an Red Cross Hospital, 710054 Xi’an, Shaanxi, PR China; 4 Department of Biosciences, Applied Biotechnology, University of Kuopio, Bioteknia 2, 70211, Kuopio, Finland

**Keywords:** JNK and p38 pathways, ATF2, Apoptosis, Chondrocytes, Cartilage, Kashin-Beck disease

## Abstract

**Background:**

The purpose of the study was to understand the function and expression of ATF2 by JNK and p38 signal pathways in the chondrocytes apoptosis of articular cartilage of the Kashin-Beck disease (KBD).

**Methods:**

The changes of ATF2, JNK and p38 mRNAs and proteins were investigated between cartilage and chondrocyte as well as KBD and normal. JNK and p38 inhibitors were used as treatments to prevent apoptosis in chondrocytes from KBD patients.

**Results:**

It was found that the protein levels of p-p38, p-JNK, ATF2 and p-ATF2 increased in KBD human cartilage which is in line with the higher mRNA levels of p38, JNK and ATF2 as compared both with normal cartilage and KBD chondrocytes. In addition, p-ATF2 was only detected in KBD cartilage. Furthermore, JNK inhibitor was more effective than p38 inhibitor in preventing chondrocyte apoptosis at equal concentrations of 10 μM.

**Conclusion:**

These findings indicated the expression of p-ATF2 by JNK and p38 signal pathways involved in the chondrocyte apoptosis in cartilage with KBD.

## Background

Kashin-Beck disease (KBD) is a chronic, endemic osteoarthropathy which occurs mainly in regions from the northeast China to Sichuan-Tibet Plateau, Russia and North Korea [1]. Approximately 0.7 million patients in China suffer from the disease, and the 105 million residents who are living in the above-mentioned provinces are at risk [2-4]. Three major environmental etiologies have been suggested: endemic selenium deficiency, cereal contamination by mycotoxin-producing fungi, and high humic acid levels in drinking water [5-11].

The KBD manifests cartilage specific pathological changes and chondrocytes apoptosis in vivo. It affects mainly the developing hyaline cartilage, causing apoptosis and necrosis of chondrocytes in the epiphyseal, growth plate and articular cartilage. Clinically, this disease manifests as enlarged interphalangeal joints, shortened fingers, deformed and enlarged joints, and limited motion of joints in the extremities. These result in stunted development of cartilage and bone, and finally secondary osteoarthropathy [12-14]. KBD patients were diagnosed by “Diagnosis criterion of Kashin–Beck disease” in China (WS/ T207—2010) [15]. For adults with KBD, the deformed joints, especially in the weight-bearing joints, keep them away from work and affect their normal lives. The current treatment for KBD patients with serious pathological characteristics (grade II/III) is to do surgery on the abnormal articular cartilage. Meanwhile, investigation of the pathology of KBD as well as the effective prevention and therapy methods are a work in progress.

c-Jun N-terminal kinase (JNK) and p38 protein kinase pathways are two major pathways of mitogen-activated protein kinase (MAPK) signal transduction [16], which play important roles in the stimulation of apoptotic signaling as well as inflammatory diseases [17-19]. The KBD is a particular type of osteoarthropathy, manifested by chondrocyte apoptosis and pathological changes after cartilage necrosis. Inflammation may be both a primary event in KBD and/or a secondary event in the disease for the observed biochemical changes with the cartilage, such as the higher expression of TNF-α, VEGF, TGF-β and IL-1 [20,21].

Our previous study comparing gene expression levels between KBD and normal articular cartilage tissue revealed a number of up-regulated genes related to apoptosis, e.g. TNFAIP6, TNFRSF11B, BCL-2, BAX and others [20]. A reanalysis of this data pointed out also p38 (NM_001315), JNK (NM_002750) and ATF2 (NM_001880) were up-regulated more than two-fold in KBD patients as compared to normal donors (p-value<0.01). Therefore, we hypothesized that JNK and p38 signaling pathways may be related to the cartilage degeneration in KBD patients.

In our work, the mRNA and protein levels of p38, JNK and ATF2 were studied between cartilage and chondrocyte as well as KBD and normal. Meanwhile, the effects of JNK and p38 inhibitors on the culture of KBD chondrocytes were determined to understand their function in the chondrocytes apoptosis of KBD.

## Methods

### Sampling of human articular cartilage

Specimens of human articular cartilage were collected from a total of 15 KBD patients (9 males and 6 females, 44-58 years of age) who were diagnosed as the second or third degree of KBD, based on the Diagnosing Criteria to Kashin-Beck disease in China [15,22]. The donors were inhabitants of endemic regions of Linyou, Yongshou, and Qianyang in Shaanxi Province of China. Normal human knee cartilage samples were collected from donors (4 males and 2 females, 38-55 years-of age) who were suffering from traffic accidents and undergoing total knee replacement surgery from non-KBD areas. The health status of control cartilage samples was examined through histological examination of hematoxylin-eosin stained sections to exclude osteoarthritis (OA), rheumatoid arthritis (RA) and other bone and cartilage diseases. Permission for this study was given by the Human Ethics Committee of the Xi’an Jiaotong University. All patients and normal donors provided a written informed consent for participation in the study and publication their individual clinical details and clinical images.

**Figure 1:**
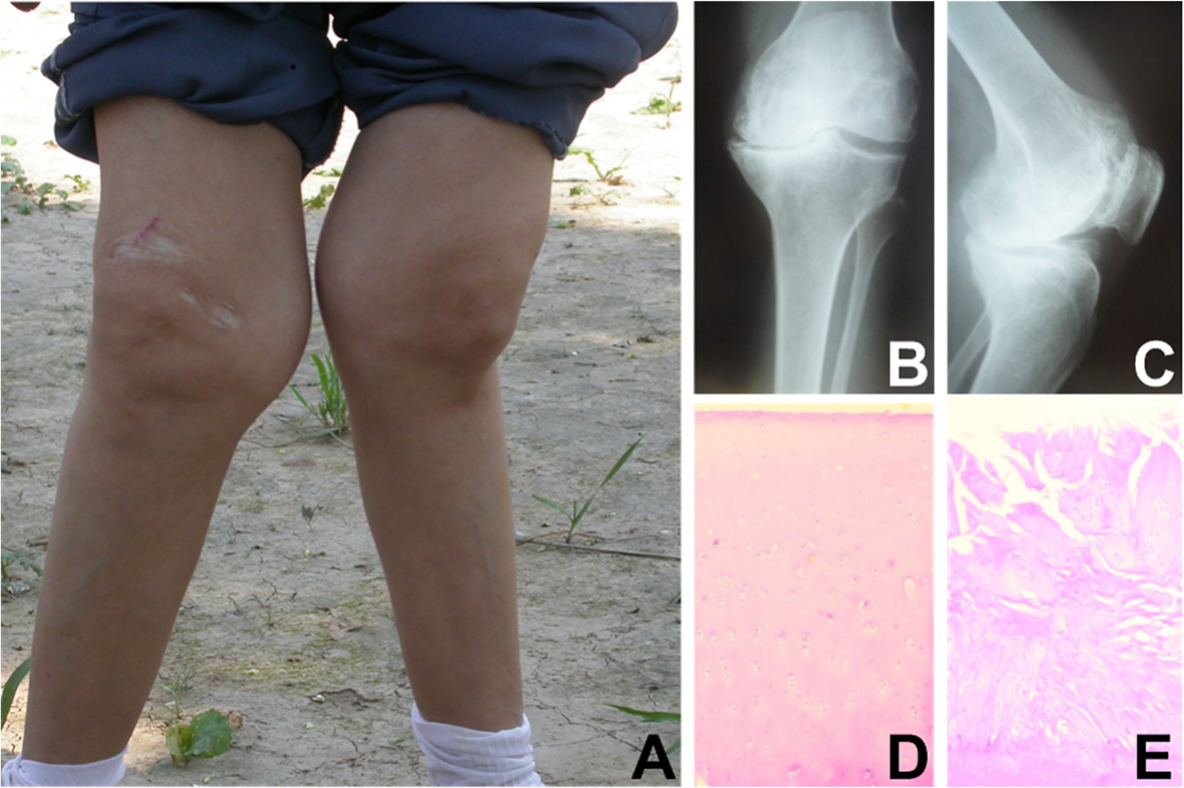
**Characteristics of KBD pateients. A.** Representative patient with grade III KBD (male patient, age 55 years), showing retarded growth of the two knees. **B** and **C.** Radiographic images of the left knee of a patient with grade II KBD (male patient, age 48 years), **(B)** anteroposterior and **(C)** lateral directions, showing an enlarged joint space and obvious joint space narrowing. **D** Hematoxylin-eosin staining of adult control articular cartilage (50 y, male), and **E** Hematoxylin-eosin staining of KBD articular cartilage with chondrocyte clusters in the eroded surface, necrotic chondrocytes and fibrous cartilage in the middle and deep layer of knee joint (55 y, male). (D and E, 40 ×).

Cartilage tissues of every KBD donor were divided into two parts, so that one part was used to extract mRNAs and proteins, while the other part was used for cell culture. One representative patient with grade III KBD, the radiographic images of the left knee of a patient with grade II KBD and HE staining pictures of articular cartilage from normal and KBD are shown in Figure 1. Meanwhile, proteins extracted from every normal donor were kept separately and the rest cartilage tissues of normal donors were used for cell culture.

### Cultivation of human articular chondrocytes

Within 8 h after surgery, the cartilage tissue was collected and washed with phosphate-buffered saline (PBS) three times, then the cartilage was cut into small pieces, which were incubated at 37°C with trypsin for 10 min. After removing the trypsin solution, the cartilage was digested at 37°C with type II collagenase using 1 ml of digestion solution per 100 mg tissue (Gibco, Grand Island, NY, USA). Every 3 hours, the digested fluid was collected through gauze to remove undigested cartilage fragments, then we collected the chondrocytes by centrifugation and reused the collagenase solution to digest the cartilage pieces. This protocol was repeated up to three times until we got enough chondrocytes for further experiments. Chondrocytes from individual donors were kept strictly separated in all experiments. Cells were plated in cell culture flasks in DMEM/F12 (1:1) (HyClone, Thermo Scientific, Logan, UT, USA), supplemented with 10% fetal bovine serum (FBS), 100 U/ml of penicillin, and 100 μg/ml of streptomycin, and maintained in a humidified atmosphere at 5% CO_2_ and 37°C. The medium was renewed two or three times a week according to the cell growth state. Primary cells were used in all experiments.

### RNA extraction and real-time quantitive PCR

Total RNAs were extracted using RNAfast200 kit (Fastagen, Shanghai, China), whose quality and concentration were assayed by a NanoDrop spectrophotometer (Thermo Scientific, USA), and then subjected to reverse transcription using RevertAidTM First Strand cDNA Synthesis kit (Fermentas, MBI, Vilnius, Lithuania) by Eppendorf gradient type mastercycler (Eppendorf, Hamburg, Germany). Reverse transcription products were used for quantitative real-time PCR analysis performed with iQTM5 Real-Time PCR Detection Systems device (Bio-Rad, Philadelphia, PA, USA) using BioEasy SYBR Green I Real Time PCR Kit (Bioer, Hangzhou, China) with oligonucleotide pairs specific for human JNK, p38, ATF2 and GAPDH with the following cycling conditions: 94°C for 2 mins, 94°C for 10 s, 58°C for 30 s, and 72°C for 30 s for 40 cycles, followed by a melting curve analysis. The forward and reverse primer pairs designed to generate a 102-bp fragment of JNK, a 107-bp fragment of p38, a 104-bp fragment of ATF2, and a 226-bp fragment of GAPDH (an internal control) are presented in Table 1. The results of relative gene expression data were analyzed using Real-Time quantitative PCR and the 2^-∆∆CT^ method [23].

**Table 1:** The primers used for the real-time reverse transcription PCR

**Gene**	**Forward primer**	**Reverse primer**
JNK		
(NM_002750)	5’- CAA GCA CCT TCA TTC TGC TG-3’	5’- GCC AGA CCG AAG TCA AGA AT-3’
p38		
(NM_001315)	5’- CGA GCG TTA CCA GAA CCT GT-3’	5’- TGG AGA GCT TCT TCA CTG CC -3’
ATF2		
(NM_001880)	5’- GGT GCT TTG TAA ACA CGG CT -3’	5’- GCA GTC CTT TCT CAA GTT TCC -3’
GAPDH	5’- GAA GGT GAA GGT CGG AGT C-3’	5’- GAA GAT GGT GAT GGG ATT TC-3’

### Western blot analysis

The proteins from cartilage and chondrocytes of KBD patients and from normal human cartilage were extracted with RIPA Cell Lysis Solution (Beyotime, Jiangsu, China), which was supplemented with PMSF and PhosSTOP alkaline phosphatase inhibitors (Hoffmann-La Roche Ltd, Basel, Switzerland). Protein concentration was assayed to adjust an equivalent loading dose of 50 μg. Before electrophoresis, all the samples were boiled in sodium dodecylsulfate (SDS) sample buffer for 5 mins. The lysates were separated by polyacrylamide gel electrophoresis and transferred to a nitrocellulose membrane (Bio-Rad, Hercules, CA, USA). The membrane was then incubated at room temperature in a blocking solution composed of 5% skimmed milk powder dissolved in Tris-buffered saline (TBS), pH 7.4, containing 0.1% Tween-20 for 1 h, followed by incubation in the blocking solution with anti-human JNK, p-JNK, p38, p-p38, ATF2, p-ATF2 antibodies separately at 4°C overnight. All the antibodies were purchased from Bioworld Technology (St. Louis Park, MN, USA; Cat No: BS3631, BS4763, BS3567, BS4766, BS1021, BS4018). The membrane was washed three times in TBS-Tween (5 mins each), and then incubated with an HRP-conjugated IgG antibody in the blocking solution. After washed three times in TBS-Tween and once in TBS (5 mins each), the immunoreactive protein was visualized using an enhanced chemiluminescence detection kit (ECL, Thermo Scientific, USA). To show equal loading of the protein samples, β-actin was used as internal control.

### The effects of JNK and p38 inhibitors on the culture of KBD chondrocytes

In order to understand if JNK and p38 activated in the apoptosis of KBD chondrocytes, we seeded primary KBD chondrocytes on 6-well dishes and divided into 3 groups: 1) control group, 2) p38 inhibitor (SB203580) group, 3) JNK inhibitor (SP600125) group. The effective inhibition doses of SB203580 for p38 and SP600125 for JNK protein with 10 μM and 20 μM were tested with KBD chondrocytes.

For control groups we follow the above mentioned chondrocytes culture protocol, while for JNK and p38 inhibitors groups, the culture medium was added with SB203580 and SP600125 individually before used for cell cultures. One week later, early chondrocyte apoptosis rates of the 3 groups were determined by a flow cytometer (Becton Dickinson, Mountain View, CA, USA) using AnnexinV-FITC Apoptosis Detection kit according to the manufacturers’ instructions (KenGEN, Nanjing, China). Moreover, apoptotic morphological changes in the nuclear chromatin were detected by DAPI stain. Chondrocytes were washed with PBS and incubation with DAPI stain solution for 10 min (Beyotime, Haimen, China). Then the chondrocytes were viewed under a fluorescence microscope (Olympus, IX-70, Japan) within 2 h. Furthermore, real-time quantitative PCR and western blot analyses were used to compare mRNA (NM_002750, NM_001315, NM_001880) and protein (p-JNK, p-p38, ATF2, p-ATF2) levels among the groups.

### Statistical analysis

Every sample was studied in triplicate. Data are presented as mean ± SD. Comparisons between the groups were carried out using the Student’s two-tailed t-test. *P*-values less than or equal to 0.05 were considered significant.

## Results

### Changes of p38, JNK and ATF2 mRNAs between KBD cartilage and chondrocyte cultures

**Figure 2:**
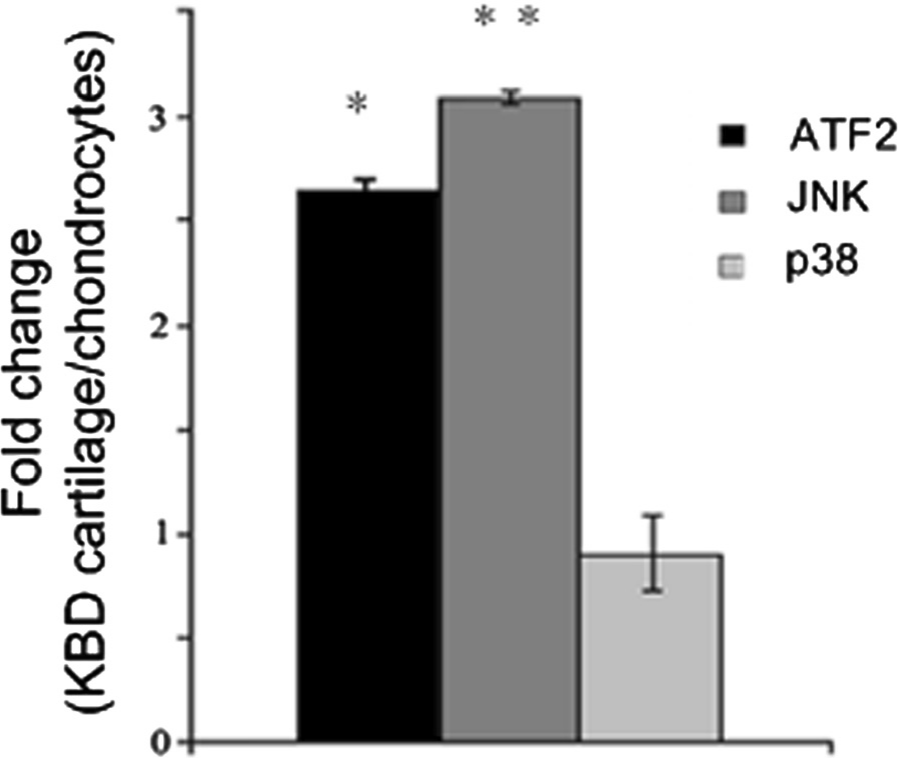
**qPCR of ATF2, JNK and p38 in cartilage compared with chondrocytes both from KBD**. The expression levels of p38, JNK and ATF2 mRNAs were about 1.3-fold, 3.2-fold and 2.7-fold higher in cartilage than in chondrocytes samples. * *p* < 0.05

Gene expression profiles may vary according to cell culture conditions. Therefore, we performed a quantitative RT-PCR analysis to compare the levels of ATF2, JNK and p38 in KBD cartilage and those in cultured KBD chondrocytes from the same donors. To our surprise, it could be observed that the expression levels of JNK and ATF2 mRNAs were about 3.2-fold (*p* < 0.01) and 2.7-fold (*p* < 0.05) higher in cartilage samples than in chondrocytes, while p38 levels remained stable with 1.3-fold in cartilage compare with chondrocytes (Figure 2).

### Changes of p-p38/p38, p-JNK/JNK and p-ATF2/ATF2 proteins

**Figure 3:**
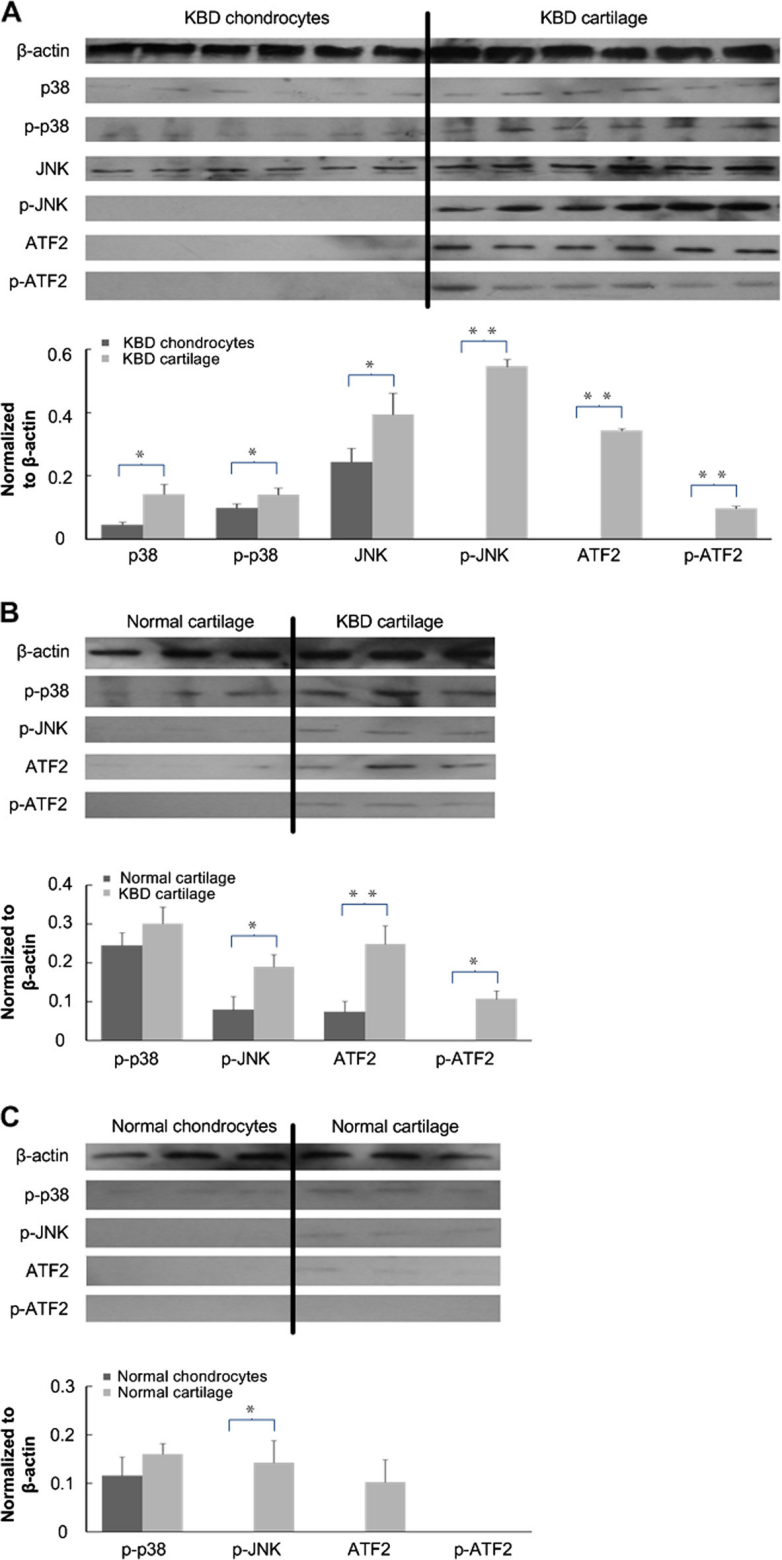
**Results of Western blotting analysis. A.** Western blotting of p38, p-p38, JNK, p-JNK, ATF2 and p-ATF2 in cartilage and chondrocytes both from KBD patient samples. p-JNK, ATF2 and p-ATF2 could not be detected in chondrocytes from KBD patients. **B.** Western blotting of p-p38, p-JNK, ATF2 and p-ATF2 in normal and KBD cartilage samples. p-ATF2 could not be detected in normal cartilage. **C.** Western blotting of p-p38, p-JNK, ATF2 and p-ATF2 in cartilage and chondrocytes both from normal samples. The levels of p-p38, p-JNK, ATF2 were higher in cartilage than cultured chondrocytes and p-ATF2 was absent in cartilage and chondrocytes. * *p* < 0.05, * * *p* < 0.01.

In order to find out whether the observed changes in mRNA levels were reflected at the protein levels, western blot analysis was performed. The protein levels of p38, p-p38 and JNK were detected with a significant increase in KBD cartilage compared with KBD chondrocytes (*p* < 0.05, and were 0.025, 0.040 and 0.032 respectively). Interestingly, the p-JNK, ATF2 and p-ATF2 could only be detected in KBD cartilage samples; the decrease in JNK and ATF2 total protein levels in cultured chondrocytes was in line with the lower level of their mRNA expression respectively (Figure 3A).

### The presence of p-p38, p-JNK, ATF2 and p-ATF2 in normal and KBD cartilage

Whether the expression of p-p38, p-JNK, ATF2, and p-ATF2 was specific to KBD was unknown, western blot analysis of samples isolated from three normal donors and three KBD patients was performed. It could be observed that only the phosphorylated ATF2 could be detected in the cartilage of KBD patients, while the expression of p-p38, p-JNK (*p* = 0.015) and ATF2 (*p* = 0.005) increased dramatically in the KBD cartilage when compared with that in normal cartilage (Figure 3B).

### The presence of p-p38, p-JNK, ATF2 and p-ATF2 in normal cartilage and chondrocyte cultures

Western blot analysis of cartilage and chondrocyte samples isolated from three normal donors was performed. The level of p-p38 in normal cartilage was a bit higher than that in normal chondrocytes. Meanwhile, the levels of ATF2 (*p* = 0.059) and phosphorylated JNK (*p* = 0.032) were detected in the cartilage samples other than in the chondrocyte samples, and phosphorylated ATF2 was absent in both cartilage and chondrocyte samples (Figure 3C).

### The effects of JNK and p38 inhibitors on the culture of KBD chondrocytes

**Figure 4:**
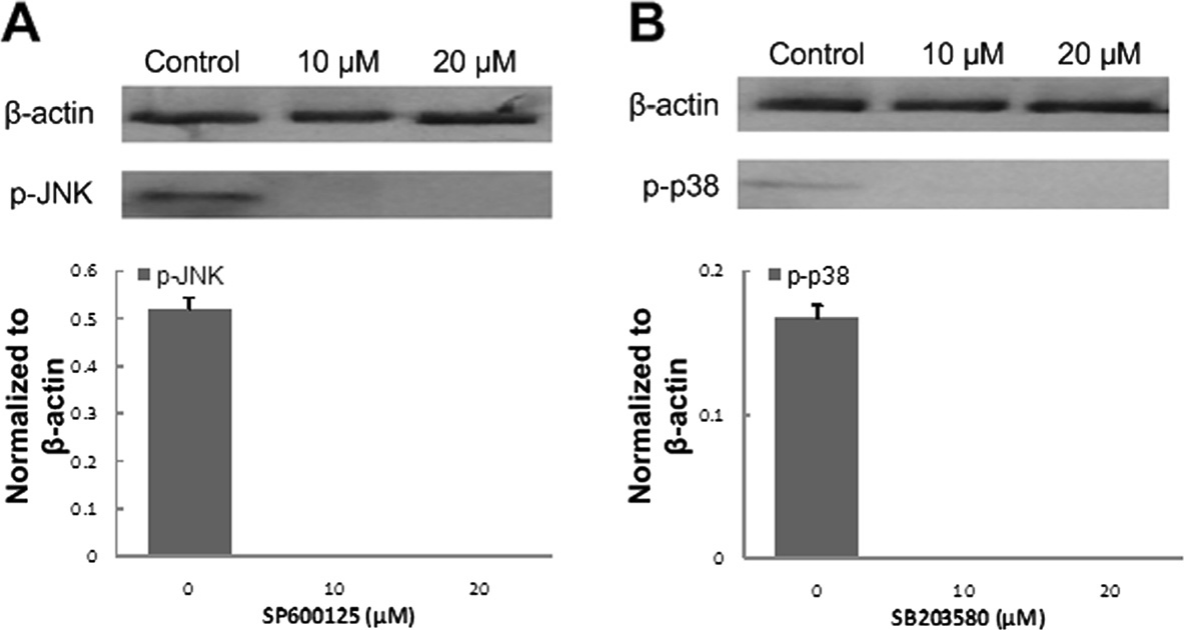
**Western blotting results of inhibitors. A.** Western blotting showed different doses of JNK inhibitor SP600125 on the expression of p-JNK. **B.** Western blotting showed different doses of p38 inhibitor SB203580 on the expression of p-p38.

Figure 4 showed that 10 μM and 20 μM for each inhibitor showed targeted phosphate protein blocked. Therefore, lower dose with 10 μM was selected as the effective inhibition dose of SB203580 for p38 and SP600125 for JNK.

**Figure 5:**
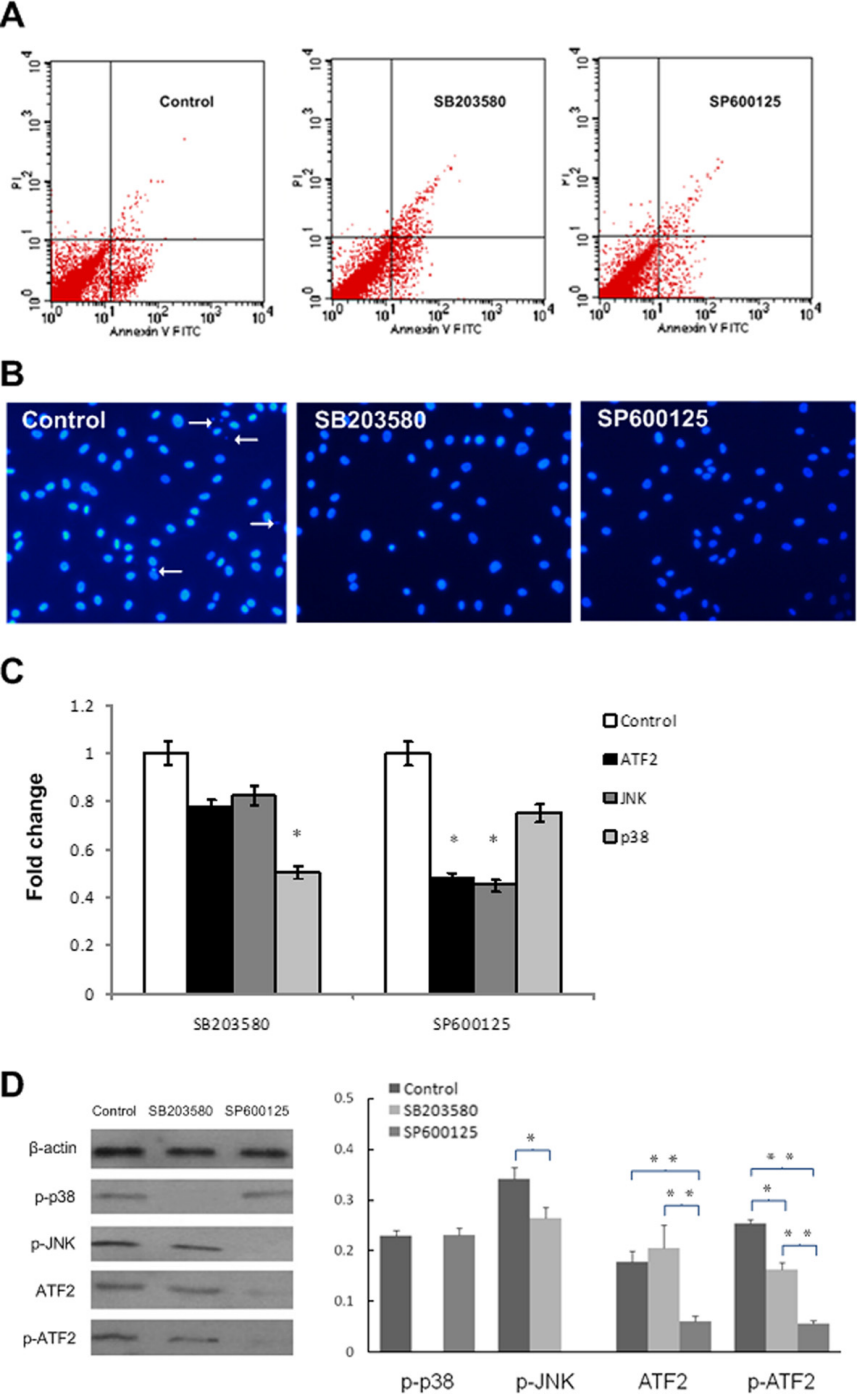
**Analysis results of different treated groups. A.** Apoptosis analysis of chondrocytes after different treatments for 3 days (control cultures with early apoptosis rate of 7.2 ± 1.3%; for cultures supplemented with p38 inhibitor SB203580 the rate was 5.8 ± 1.4%; those supplemented with JNK inhibitor SP600125 was 3.4 ± 1.1%). **B.** DAPI stain images of KBD chondrocytes in different groups (200 ×, control, SB203580 and SP600125, control groups showed the nuclear fragmentation and condensation). **C.** qPCR of ATF2, JNK & p38 in chondrocytes of different treatment normalized to GAPDH and normalized to control. SP600125 and SB203580 decreased the fold changes of ATF2 to 0.5 and 0.8 respectively, p < 0.001. **D.** Western blotting of p-p38, p-JNK, ATF2 and p-ATF2 in chondrocytes with different treatment. (The levels of p-p38, p-JNK, ATF2 and p-ATF2 were lower in chondrocytes cultured with inhibitors, and p-ATF2 was more likely to be influenced by JNK inhibitor). * *p* < 0.05, * * *p* < 0.01.

In control group, the early apoptosis rate was 7.2 ± 1.3% (Figure 5A). The JNK inhibitors SP600125 decreased apoptosis rate from 7.2 ± 1.3% to 3.4 ± 1.1% (*p* < 0.05), while p38 inhibitor SB203580 had a less pronounced effect on the percentage of apoptosis rate (5.8 ± 1.4%).

As shown in Figure 5B, DAPI-stained nuclei from chondrocytes of control group showed cytoplasmic signs of apoptotic cell death, meanwhile, the nuclear fragmentation and condensation were evident. In contrast, KBD nuclei were bigger and rounder with inhibitors.

The expression levels of the p38, JNK and ATF2 mRNA were higher in the control group (Figure 5C). The addition of SP600125 reduced the expression of JNK and ATF2 mRNAs to less than 0.5-fold each (*p* < 0.05); SB203580 decreased the level of p38 mRNAs to about 0.5-fold as well while had a less effect on the level of ATF2 mRNA to 0.8-fold.

The p38 inhibitor SB203080 specifically prevented phosphorylation of p38 (Figure 5D), while affected slightly to p-JNK, ATF2 and p-ATF2; meanwhile, the JNK inhibitor SP600125 efficiently blocked the phosphorylation of JNK and the total protein level of ATF2 as well as p-ATF2. SP600125 was not only a potent inhibitor of ATF2 phosphorylation; it also decreased the protein amount of ATF2, which was in line with its mRNA expression.

## Discussion

The pathological mechanisms related to KBD are poorly understood. One of the hallmarks of the disease is the apoptosis of chondrocytes. Signaling pathways of JNK and p38 have been associated with apoptotic events, which could be linked to KBD. However during the cell culture procedure, we had to wait for at least two weeks until we could get enough chondrocytes, meanwhile only the survival chondrocytes were examined, and thus we might miss important information of certain mRNAs and proteins in the two pathways. In this article, their possible association with KBD is studied and demonstrated by comparing mRNAs and proteins related to these pathways between cartilage and chondrocyte as well as KBD and normal, and the JNK and p38 inhibitors were used to investigate their function in the KBD chondrocyte apoptosis.

As shown, JNK and ATF2 mRNAs were higher expressed in KBD cartilage samples than normal samples by reanalysis of previous data. While kept in cell culture conditions, the KBD chondrocytes had their expression declined significantly. Additionally, total proteins of p-JNK, ATF2 and p-ATF2 were less expressed in normal cartilage samples and after cell culture they were non-detectable in KBD and normal chondrocytes. To conclusion, p-ATF2 was not observed in normal cartilage, and after cell culture, p-JNK, ATF2 and p-ATF2 were not detected in all the chondrocytes samples from KBD and normal, which indicated that p-ATF2 could only be detected in KBD cartilage. The phenomena may refer to the reason why the KBD chondrocyte could survive better in cell culture medium other than the serum from KBD patients. The previous studies have shown the increased levels of TNF-α and IL-1β, abnormal expression of Bax and Bcl-2 in the serum and synovial fluid of KBD patients [24]. Moreover elevated levels of many apoptotic markers [25] and NO level in serum [26] have been shown in KBD, which may lead to the stimulated expression of p-ATF2, and if we use the serum from KBD patients to culture normal rabbit chondrocyte, it would result in the pathological changes of normal chondrocytes similar to KBD chondrocytes [27,28].

ATF2 is normally activated in response to signals that converge on stress-activated protein kinases p38 and JNK [29]. While during the study of KBD chondrocytes cultured individually with p38 and JNK inhibitors, we observed that the early apoptosis rates of KBD chondrocytes decreased by using both of them, and JNK inhibitor could better alleviate KBD apoptosis than p38 inhibitor (3.4 ± 1.1% vs 5.8 ± 1.4%), meanwhile JNK inhibitor showed ATF2 and p-ATF2 protein partially blocked in KBD chondrocytes better than p38 inhibitor, which was accordance with the decreased expression of ATF2 mRNA level. The results suggested the expression of p-ATF2 in the apoptotic KBD chondrocyte was mainly through JNK signal pathway.

JNK and p38 MAPK pathways are activated by stress and inflammatory signals with a variety of cell receptors, including death receptors (FAS), inflammatory cytokine receptors (TNF-a, TGF-b), G protein-coupled receptors and antigen receptors [30-32]. Selenium deficiency induced dysfunction of selenoproteins which exhibited a variety of biological functions, including antioxidant functions, maintaining cellular redox balance, and heavy metal detoxification [33,34]; therefore compromise of such important proteins would lead to oxidative stress and apoptosis. T-2 toxin was also shown to induce reactive oxygen species and compromise mitochondrial oxidative phosphorylation, which lead to mitochondria-mediated apoptosis [35,36]. In a rat model, T-2 toxin together with selenium deficiency has been reported to cause chondronecrosis development with similarities to KBD [37]. Whether JNK and p38 pathways involved in the chondrocytes apoptosis induced by T-2 toxin was under way to clarify. Meanwhile, we will pay close attention to selenium, especially the mechanism of the inhibition effect of nano-selenium chondroitin sulfate [38] which was prepared by our group on the T-2 toxin induced apoptosis in the later experiments.

**Figure 6:**
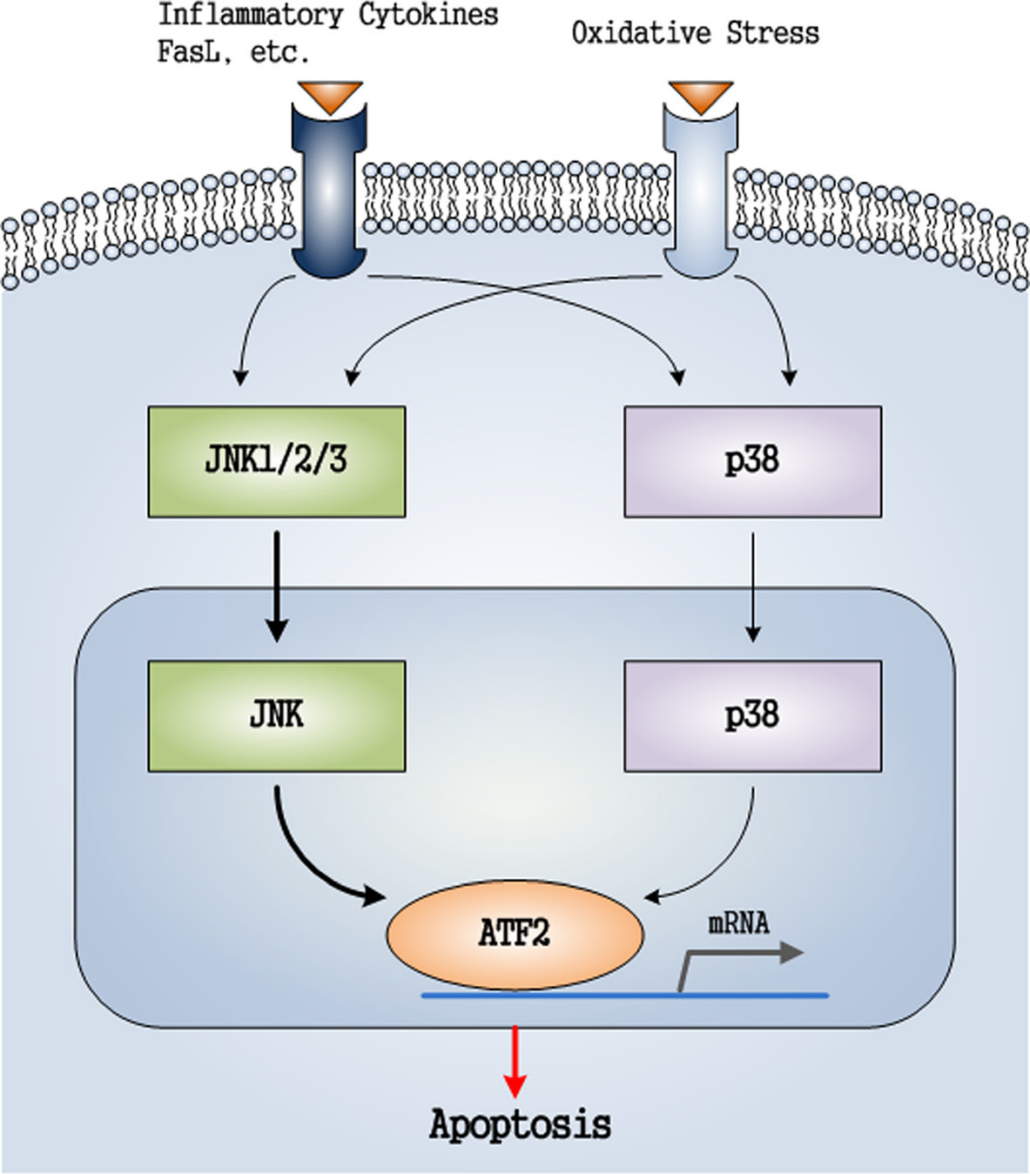
Supposed functional factors in JNK and p38 pathways related to chondrocytes apoptosis of cartilage with KBD.

Based on the above, a supposed pathological process for KBD (Figure 6) is as follows: A signal (inflammatory factor or oxidative stress) arrives at the cell surface, activates the corresponding receptor that leads to the production of a second messenger, such as cAMP or Ca^2+^, which in turn activates JNK/p38 protein kinase. The process could be described as: FasL, inflammatory cytokines, oxidative stresses → JNK/p38 → ATF2, after which ATF2 translocates to the cell nucleus, where it activates and regulates the expression of selected genes. Finally, the activated JNK/p38 pathway induces chondrocyte apoptosis. More details of possible functions of JNK/p38 signal pathway in KBD, and the comparison of KBD with osteoarthritis, need to be examined in further studies.

In summary, obvious higher mRNA levels of JNK and ATF2 as well as elevated expression levels of p-JNK, p-ATF2 and ATF2 were observed in KBD cartilage. In addition, JNK inhibitor was more effective than p38 inhibitor in the prevention of KBD chondrocyte apoptosis. The current findings suggest the involvement of JNK and p38 pathways correlated factors in the chondrocytes apoptosis of KBD cartilage.

## Conclusion

In conclusion, our studies have shown that the expression of p-ATF2 is important for the apoptosis of chondrocytes in cartilage with KBD. The apoptotic function of ATF2 in KBD cartilage is mainly enhanced through JNK signal pathway other than p38 signal pathway. Thus, the activation of ATF2 through JNK pathway may play an important role in chondrocyte apoptosis of human KBD, which maybe a possible target in the prevention and treatment of KBD.

## Abbreviations

KBD: Kashin-Beck disease; OA: Osteoarthritis; RA: Rheumatoid arthritis; MAPK: Mitogen-activated protein kinase; JNK: c-Jun N-terminal kinase; ATF2: Activating transcription factor 2; IL-1: Interleukin-1; IL-1β: Interleukin-1β; TNF-α: Tumor necrosis factor-α; VEGF: Vascular endothelial growth factor; TGF-β: Transforming growth factor-β; TNFAIP6: Tumor necrosis factor alpha-induced protein 6; TNFRSF11B: Tumor necrosis factor receptor superfamily member 11B; BCL-2: B-cell lymphoma/leukemia-2; BAX: The Bcl-2–associated X protein; FBS: Fetal bovine serum; SDS: Sodium dodecylsulfate; TBS: Tris-buffered saline; PBS: phosphate buffer; MTT: 3-(4,5)-dimethylthiahiazo(-z-y1)-3,5-di- phenytetrazo -liumromide; FCM: Flow cytometer; FAS: Fatty acid synthase; FASL: FAS ligand; CRE: cAMP-responsive element; CREB: cAMP-response element binding protein; CBP: CREB-binding protein.

## Competing interests

The authors declare that they have no competing interests.

## Authors’ contributions

Miss H had full access to all of the data in the study and takes responsibility for the integrity of the data and accuracy of the data analysis. Study design: HG. Acquisition of data: H, W, Z. Analysis and interpretation of data: H, G, L, T. Manuscript preparation: H, G, L. Sample collection: H, X, L. All authors read and approved the final manuscript.

## Acknowledgements

This work was supported by the National Natural Scientific Foundation of China (grant no. 30972556) and the Specialized Research Fund for the Doctoral Program of Higher Education of China (grant no. 20090201110049), and the Academy of Finland (grant no.128117).We thank the orthopedic surgeons and nursing staff of the Department of Orthopaedics or Trauma in the Xi’an Red Cross Hospital, the Second Affiliated Hospital of Xian Jiaotong University and Shaanxi Endemic Disease Hospital for support and cooperation in the collection of cartilage specimens.

## Pre-publication history

The pre-publication history for this paper can be accessed here:

http://www.biomedcentral.com/1471-2474/14/209/prepub
